# Suboptimal Greedy Power Allocation Schemes for Discrete Bit Loading

**DOI:** 10.1155/2013/370261

**Published:** 2013-12-31

**Authors:** Waleed Al-Hanafy, Stephan Weiss

**Affiliations:** ^1^Electronics and Communication Engineering Department, Faculty of Electronic Engineering, Menoufia University, Menouf 32952, Egypt; ^2^Centre for White Space Communications/CeSIP, Department of EEE, University of Strathclyde, Glasgow G1 1XW, Scotland, UK

## Abstract

We consider low cost discrete bit loading based on greedy power allocation (GPA) under the constraints of total transmit power budget, target BER, and maximum permissible QAM modulation order. Compared to the standard GPA, which is optimal in terms of maximising the data throughput, three suboptimal schemes are proposed, which perform GPA on subsets of subchannels only. These subsets are created by considering the minimum SNR boundaries of QAM levels for a given target BER. We demonstrate how these schemes can significantly reduce the computational complexity required for power allocation, particularly in the case of a large number of subchannels. Two of the proposed algorithms can achieve near optimal performance including a transfer of residual power between subsets at the expense of a very small extra cost. By simulations, we show that the two near optimal schemes, while greatly reducing complexity, perform best in two separate and distinct SNR regions.

## 1. Introduction

In OFDM, multiplexing over multiple-input multiple-output (MIMO) channels, or general transmultiplexing techniques, a number of independent subcarriers or subchannels arise for transmission,which differ in SNR. Maximising the channel capacity or data throughput under the constraint of limited transmit power leads to the well-known and simple water-filling algorithm [1]. Water-filling is generally followed by bit loading, where *b*_*i*_ bits are allocated to the QAM symbols transmitted over the *i*th subchannel. To achieve an identical target bit error ratio (BER) across all subchannels leads to *b*_*i*_ ∈ ℝ, which needs to be rounded off to the nearest integer *b*_*i*_^(r)^ = ⌊*b*_*i*_⌋, thus lowering the overall throughput. Furthermore, unbounded modulation orders *b*_*i*_^(r)^ → *∞* in the case of infinite SNR are required to efficiently utilise the transmit power but are practically unfeasible.

In order to optimise capacity and throughput, a wide range of methods has been suggested in the literature. Pure water-filling-based solutions have been reported in [2–4], leading to some of the above stated problems. Reallocation of the excess power when realising the target BER given *b*_*i*_^(r)^ ∈ *ℤ* and the SNR in the *i*th subchannel has led to a rate-optimal algorithm known as the greedy algorithm [5, 6], of which a number of different variations have emerged constraining either the average BER [7] or the total power [8]. For a good review of greedy algorithms, please refer to [9]. Owing to the iterative nature of these algorithms to optimally achieve their respective objective functions, the computational complexity dramatically increases with the number of subchannels. The situation becomes practically prohibitive for multicarrier systems (such as OFDM) as the number of subcarriers is usually high and can reach, for example, up to 2^13^ for digital video broadcasting (DVB) for terrestrial (DVB-T) or handheld (DVB-H) applications [10–12].

While achieving rate optimality, the family of greedy algorithms is also known to be greedy in terms of computing requirements. Therefore, reduced complexity schemes are either water-filling-based only [2] or aim at simplifications [13]. In this paper we propose a novel suboptimal greedy algorithm, whereby the power reallocation is performed in subsets of the subchannels. We show that with simple overall power redistribution between groups, two different methods in terms of approximate overall optimisation can be proposed. These suboptimal schemes, while greatly simplifying complexity, hardly sacrifice any performance compared to the full GPA algorithm, provided that the proper algorithmic version is selected for specific SNR regions.

Different from our previous work in [14], the interest of this paper is focusing on the simplification achievements of our proposed power allocation scheme compared to the standard greedy approach by further elaborating on the complexity analysis of both algorithms. Moreover, results for multicarrier systems are included which highlight the significant reduction in complexity gained by our approach. The rest of the paper is organised as follows. In Section 2, the standard greedy approach is first reviewed including the initialisation step of uniform power allocation (UPA). Our proposed reduced-complexity schemes are presented in Section 3, where computational complexity is analysed and evaluated in Section 4. Simulation results are discussed in Section 5 and conclusions are drawn in Section 6.

## 2. The Greedy Approach

In this section, the greedy approach for the power allocation problem to maximise the transmission rate over a multichannel system is introduced.

### 2.1. Constrained Optimisation Problem

We are interested in the problem of maximising the transmission rate over a multichannel system. This problem could arise from any transmultiplexed communications system, such as narrowband MIMO systems decoupled by an SVD for precoding and equalisation [15]. Given an *N*_*R*_ × *N*_*T*_ narrowband MIMO system with *N*_*R*_ receive and *N*_*T*_ transmit antennas, the channel can be characterised by a matrix **H** ∈ *ℂ* ^*N*_*R*_×*N*_*T*_^ of complex coefficients *h*_*ij*_ which describe the gains between the *j*th transmit to the *i*th receive antennas. The singular value decomposition (SVD) in this case can be used to decouple the system **H** into *N* = rank⁡(**H**) ≤ min⁡(*N*_*T*_, *N*_*R*_) subchannels whose gains are equal to the singular values *σ*_*i*_, *i* = 1,…, *N*, that are ordered such that *σ*_*i*_ ≥ *σ*_*i*+1_  for all *i*. This is likely to result in different SNRs for each subchannel, and if all subchannels are allocated the same number of bits and transmit power, the overall system performance will be dominated by the worst subchannel with gain *g*_*N*_.

Another popular multiplex system is either SISO or MIMO OFDM. Without loss of generalisation, in the following we assume a SISO OFDM system, whereby the ISI channel is characterised by an FIR vector **h** = [*h*_0_ ⋯ *h*_*L*_] ∈ *ℂ* ^*L*+1^ of order *L*. If this OFDM system is based on an *N*-point discrete Fourier transform (DFT), then the resulting *N* subcarriers experience different gains *g*_*i*_,  *i* = 1 ⋯ *N*, that represent the Fourier coefficients of the channel impulse response; that is, *g*_*i*_ = ∑_*l*=0_^*L*−1^ *h*_*l*_ *e* ^*j*2*πil*/*N*^. The *i*th subcarrier with gain *g*_*i*_ will be used to transmit *b*_*i*_ bits per symbol.

In both cases considered previously, *N* independent subcarriers or subchannels arise, whereby in the following we will use both terms synonymously. To optimise the data throughput across such a system with *N* independent subchannels, in this paper we consider the maximisation of the sum-rate

(1) $$\begin{array}{c} \underset{}{\max}\overset{N}{\underset{i=1}{\sum }}b_{i}, \end{array}$$

constrained by the total power budget, the target bit error ratio (BER), and the maximum permissible QAM modulation order. These constraints can be formulated as

(2) $$\begin{array}{c} \overset{N}{\underset{i=1}{\sum }}P_{i}\leq P_{\text{budget}},\\ {𝒫}_{b,i}={𝒫}_{b}^{\text{target}},\;b_{i}\leq b^{\max},\;\forall i,\;1\leq i\leq N, \end{array}$$

where *P*_*i*_ is the amount of power allocated to the *i*th subchannel to achieve a BER *𝒫*_*b*,*i*_ and *b* ^max⁡^ is the maximum number of permissible bits allocated to a subchannel. Note that the target BERs are assumed to be equal, that is, *𝒫*_*b*,*i*_ = *𝒫*_*b*_^target^ in (2) for all subchannels *i* = 1,…, *N*, and therefore the subscript *i* will be dropped from the BER notation, that is, *𝒫*_*b*,*i*_ = *𝒫*_*b*_.

The channel-to-noise ratio of the *i*th subchannel can be defined as

(3) $$\begin{array}{c} \text{CN}{\text{R}}_{i}=\frac{g_{i}^{2}}{{𝒩}_{0}}, \end{array}$$

where *𝒩*_0_ is the total noise power at the receiver, whereas the SNR of this subchannel is

(4) $$\begin{array}{c} \gamma_{i}=P_{i}\times \text{CN}{\text{R}}_{i}. \end{array}$$

We consider rectangular *M*-QAM modulation of order *M*_*k*_, 1 ≤ *k* ≤ *K*, where *M*_*K*_ is the maximum QAM constellation, that is, permissible by the transmission system, that is, *M*_*K*_ = 2^*b*^max⁡^^. The BER of this modulation scheme is given by [16]

(5) 𝒫b={Q(2γi)for  BPSK,1−[1−2(1−1/Mk)Q(3γi/(Mk−1))]2log2Mkfor  M-QAM.

Assuming availability of channel state information (CSI) at the transmitter, symbols of *b*_*k*_-bits, *b*_*k*_ = log_2_ *M*_*k*_ can be loaded to a subcarrier with minimum required SNR to achieve *𝒫*_*b*_^target^ obtained from (5) as

(6) γkQAM={12[Q−1(𝒫btarget)]2for  BPSK,Mk−13[Q−1(1−1−𝒫btarget·log2Mk2(1−1/Mk))]2for  M-QAM,

where *Q* ^−1^ is the inverse of the well-known *Q* function

(7) $$\begin{array}{c} Q\left(x\right)=\frac{1}{\sqrt{2\pi }}\overset{\infty }{\underset{x}{\int }}e^{-u^{2}/2}du. \end{array}$$

Based on (6), the bit loading problem is solved in two steps—(i) a uniform power allocation (UPA) initialisation step and (ii) the greedy algorithm step—which are both described below.

### 2.2. Subchannel Grouping and UPA Algorithm

The uniform power allocation is performed by the following steps.

**Figure 1:**
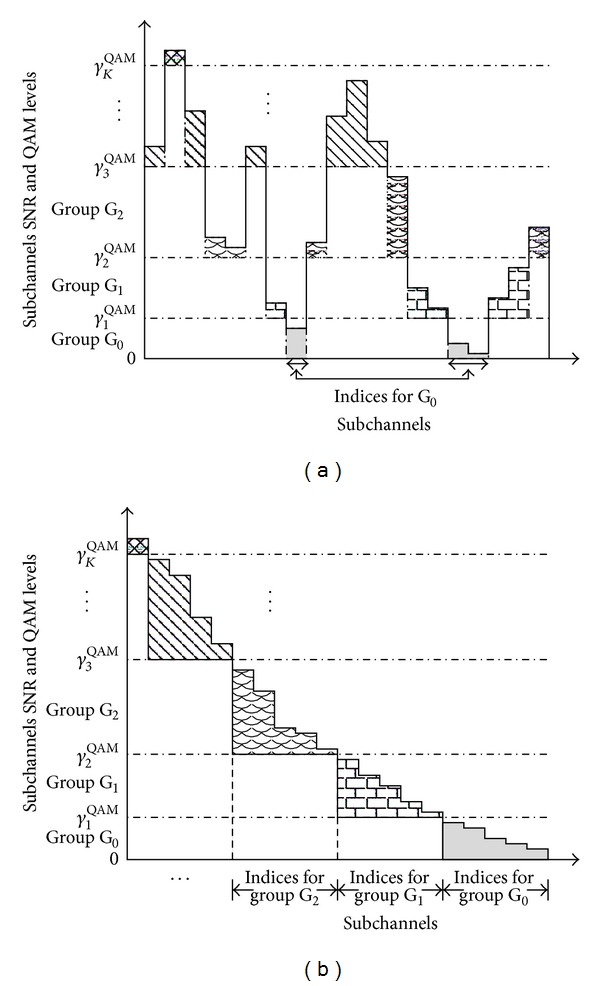
Subchannel grouping into *K* + 1 QAM groups based on their SNRs in (8) and step (3) of Section 2.2 for (a) a multicarrier system and (b) an ordered multicarrier system or a SVD-based MIMO system.

(1) Calculate *γ*_*k*_^QAM^ for all *M*_*k*_, 1 ≤ *k* ≤ *K*, and *𝒫*_*b*_ = *𝒫*_*b*_^target^ using (6).
(2) Equally allocate *P*_budget_ among all subchannels 1 ≤ *i* ≤ *N*:
  (8) $$\begin{array}{c} \gamma_{i}=P_{i}\times \text{CN}{\text{R}}_{i}=\frac{P_{\text{budget}}}{N}\times \text{CN}{\text{R}}_{i}. \end{array}$$
(3) Allocate subchannels according to their SNR *γ*_*i*_ to QAM groups G_*k*_, 0 ≤ *k* ≤ *K*, bounded by QAM levels *γ*_*k*_^QAM^ and *γ*_*k*+1_^QAM^ with *γ*_0_^QAM^ = 0 and *γ*_*K*+1_^QAM^ = +*∞* (cf.  Figure 1) such that
  (9) $$\begin{array}{c} \gamma_{i}\geq \gamma_{k}^{\text{QAM}},\;\;\gamma_{i}<\gamma_{k+1}^{\text{QAM}}. \end{array}$$
(4) For each group G_*k*_, load subchannels within this group with QAM constellation *M*_*k*_ and compute the group's total allocated bits:
  (10) $$\begin{array}{c} B_{k}^{u}=\underset{i\in {\text{G}}_{k}}{\sum }b_{i,k}^{u}=\underset{i\in {\text{G}}_{k}}{\sum }\text{lo}{\text{g}}_{2}M_{k}, \end{array}$$

with *B*_0_^*u*^ = 0. It is clear at this point and from step (3) that subchannels are resided into QAM groups of SNR levels that are below their actual SNRs, *γ*_*i*_ ≥ *γ*_*k*_^QAM^, therefore leaving some unused (excess) power:

(11) Pkex=∑i∈Gkγi−γkQAMCNRi=∑i∈GkPi−γkQAMCNRi=NkPbudgetN−∑i∈GkγkQAMCNRi,

where *N*_*k*_, 1 ≤ *k* ≤ *K*, is the number of subchannels that occupies the QAM group G_*k*_.

(5) Overall, the allocated bits and the used power for the uniform power allocation scheme are therefore

(12a) $$\begin{array}{c} B_{u}=\overset{K}{\underset{k=1}{\sum }}B_{k}^{u}, \end{array}$$

(12b) $$\begin{array}{c} P_{u}^{\text{used}}=P_{\text{budget}}-P^{\text{ex}}, \end{array}$$

where *P* ^ex^ = ∑_*k*=0_^*K*^ *P*_*k*_^ex^ is the total excess power that remains unallocated under the UPA scheme.

### 2.3. Full Greedy Power Allocation (GPA) Algorithm

The second step towards the GPA is described next. Based on the initialisation step described in the UPA,the full GPA algorithm [8] performs an iterative redistribution of the unallocated power of the UPA algorithm *P* ^ex^ by applying the algorithmic steps detailed in Algorithm 1. At each iteration, this algorithm tries to increase bit loading by upgrading (to the next higher QAM level) the subchannel with the least power requirements through an exhaustive search by performing step (4) in Algorithm 1 for all subchannels *N*. When either (i) the remaining power cannot support any further upgrades or (ii) all subchannels appear in the highest QAM level *K*, the algorithm stops, resulting in the bit allocation and power usage given by, respectively,

(13a) $$\begin{array}{c} B_{\text{gpa}}=\overset{N}{\underset{i=1}{\sum }}b_{i}^{\text{gpa}}, \end{array}$$

(13b) $$\begin{array}{c} P_{\text{gpa}}^{\text{used}}=P_{\text{budget}}-P_{d}^{\text{gpa}}\;. \end{array}$$

## 3. Proposed Low-Cost GPA Schemes

Given *B*_*k*_^*u*^ as defined in (10) and *P*_*k*_^ex^ in (11), three low-cost greedy algorithms are proposed to efficiently utilise the total excess power of the uniform power allocation in (12b) using the QAM grouping concept. More precisely, GPA is separately accomplished for each QAM group G_*k*_ aiming to increase the total bit allocation to this group and therefore the overall allocated bits. Based on the way of utilising *P*_*k*_^ex^, we propose three different algorithms, which below are referred to as (i) grouped GPA (g-GPA), (ii) power moving-up GPA (Mu-GPA), and (iii) power moving-down GPA (Md-GPA).

### 3.1. Grouped GPA (g-GPA) Algorithm

As discussed in Section 2, optimum discrete bit loading constrained by total power and maximum permissible QAM order can be performed by the GPA approach. However, the direct application of the GPA algorithm is computationally very costly due to the fact that at each iteration an exhaustive sorting of all subchannels *N* is required as evident from Algorithm 1.

A simplification of the GPA algorithm can be achieved if subchannels are first divided into QAM groups G_*k*_, 0 ≤ *k* ≤ *K*, according to their SNRs as shown in Figure 1(a). After subchannel ordering or due to the implicit ordering of the singular values in case of SVD-based decoupling of MIMO systems, the grouping as shown in Figure 1(b) arises. The GPA algorithm is therefore independently applied to each group G_*k*_, trying to allocate as much of the excess power *P*_*k*_^ex^ within this QAM group as possible. This excess power is iteratively allocated to subchannels within this group according to the greedy concept with the aim of upgrading as many subchannels as possible to the next QAM level.

The pseudocode of the g-GPA algorithm for the *k*th QAM group *G*_*k*_ is given in Algorithm 2.

Note that, different from the standard GPA, this algorithm permits upgrades to the next QAM level only for a given QAM group, with *P*_*j*_^up^ set to +*∞* in steps (5) and (6) in Algorithm 2. Therefore, some left-over (LO) power *P*_*k*_^LO^ may remain for each QAM group G_*k*_, resulting in a total LO power

(14) $$\begin{array}{c} P_{\text{g}}^{\text{LO}}=\overset{K-1}{\underset{k=0}{\sum }}P_{k}^{\text{LO}}+P_{K}^{\text{ex}}. \end{array}$$

Intuitively, for the overall performance of the g-GPA algorithm, the algorithm in Algorithm 2 has to be executed *K* times, once for each QAM group from *G*_0_ to *G*_*K*−1_, resulting in the system achieving the following bit allocation and power usage, respectively:

(15a) $$\begin{array}{c} B_{\text{g}}=\overset{K-1}{\underset{k=0}{\sum }}B_{k}^{\text{g}}+B_{K}^{u}, \end{array}$$

(15b) $$\begin{array}{c} P_{\text{g}}^{\text{used}}=P_{\text{budget}}-P_{\text{g}}^{\text{LO}}. \end{array}$$

### 3.2. Power Moving-Up GPA (Mu-GPA) Algorithm

**Figure 2:**
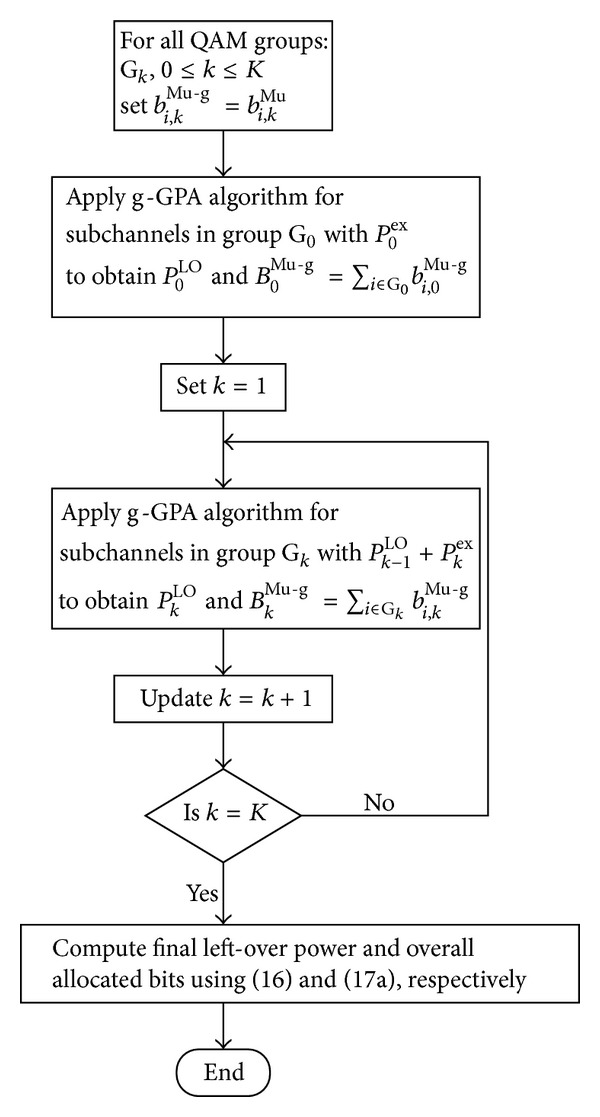
Flowchart of the Mu-GPA algorithm.

**Figure 3:**
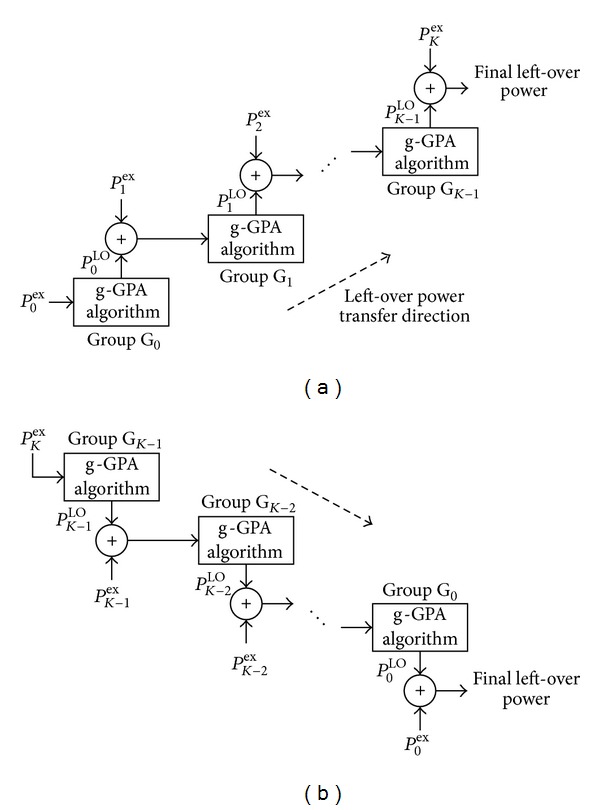
Algorithmic arrangements for (a) Mu-GPA algorithm and (b) Md-GPA algorithm with final left-over powers given, respectively, in (16) and (18).

The g-GPA algorithm results in unused power *P*_*k*_^LO^ for each QAM group. This residual power can be exploited in a second stage, whereby we first proposed to move power upwards starting from the lowest QAM group, as outlined in Figure 3(a) and by the flowchart in Figure 2. This modifies the g-GPA algorithm by considering the LO power *P*_0_^LO^ of the QAM group G_0_ after running the g-GPA algorithm on that group and assigns this power for redistribution to group G_1_. Any LO power after running g-GPA on G_1_ is then passed further upwards to G_2_ and so forth. At the *k*th algorithmic iteration, the Mu-GPA algorithm is working with G_*k*_ and tries to allocate the sum of the excess power missed by the UPA algorithm of that group as well as the LO power of the application of the g-GPA algorithm to the previous group G_*k*−1_, that is, *P*_*k*_^ex^ + *P*_*k*−1_^LO^ (cf. Figure 3(a)). Finally, the LO power resulting from the QAM group G_*K*−1_ is added to the excess power of the *K*th QAM group *P*_*K*_^ex^ to end up with a final LO power

(16) $$\begin{array}{c} P_{\text{Mu-g}}^{\text{LO}}=P_{K-1}^{\text{LO}}+P_{K}^{\text{ex}}. \end{array}$$

The overall number of allocated bits and the amount of used power for Mu-GPA are, respectively,

(17a) $$\begin{array}{c} B_{\text{Mu-g}}=\overset{K-1}{\underset{k=0}{\sum }}B_{k}^{\text{Mu-g}}+B_{K}^{u}, \end{array}$$

(17b) $$\begin{array}{c} P_{\text{Mu-g}}^{\text{used}}=P_{\text{budget}}-P_{\text{Mu-g}}^{\text{LO}}. \end{array}$$

### 3.3. Power Moving-Down GPA (Md-GPA) Algorithm

A second algorithm is proposed to exploit the residual power *P*_*k*_^LO^ of each QAM group but in a reverse direction compared to the Mu-GPA algorithm of Section 3.2. Starting from the highest-indexed QAM group G_*k*−1_ downwards to the lowest-indexed QAM group G_0_, the Md-GPA algorithm, similar to the Mu-GPA algorithm, tries to improve the bit allocation by efficiently utilising *P*_*k*_^LO^, *K* − 1 ≥ *k* ≥ 1, plus the excess power *P*_*K*_^ex^. These procedures are illustrated in Figure 3(b) which show the direction of the LO power flow. Proceeding downwards, at the *k*th stage the Md-GPA scheme applies the g-GPA algorithm for the available power that comprises both the excess power missed by the UPA algorithm of the previous QAM group (G_*k*+1_ in this case) and the LO power of the previous stage, that is, *P*_*k*+1_^ex^ + *P*_*k*+1_^LO^. Therefore, the excess power of the QAM group under consideration along with its LO power is not utilised within this group but is transferred to the next working group. This will finally result in a LO power of

(18) $$\begin{array}{c} P_{\text{Md-g}}^{\text{LO}}=P_{0}^{\text{LO}}+P_{0}^{\text{ex}}. \end{array}$$

The overall number of allocated bits and the amount of used power for Md-GPA are, respectively,

(19) $$\begin{array}{c} B_{\text{Md-g}}=\overset{K-1}{\underset{k=0}{\sum }}B_{k}^{\text{Md-g}}+B_{K}^{u}, \end{array}$$

(20) $$\begin{array}{c} P_{\text{Md-g}}^{\text{used}}=P_{\text{budget}}-P_{\text{Md-g}}^{\text{LO}}. \end{array}$$

## 4. Computational Complexity Evaluation

In order to address the significance of the proposed power loading schemes in terms of simplicity compared to the full GPA algorithm, the computational complexity of both g-GPA and GPA algorithms is evaluated. Instead of jointly applying the GPA algorithm across all subchannels which consequently requires high system complexity especially for large numbers of subchannels, the g-GPA algorithm only addresses a subset of subchannels within a specific QAM group at a time. Beyond the effect of the QAM grouping concept, a further reduction in complexity can be achieved if subchannels are ordered with respect to their gains CNR_*i*_, as found with SVD-based decoupling of MIMO systems. In this case, search step (3) in Algorithm 2 can be replaced by a simple incremental indexing.

Referring to Algorithms 1 and 2, the computational complexities of both GPA and g-GPA algorithms are summarised in Table 1, whereby the number of operations (NoO) is computed for each algorithm. We consider the cases where subchannel SNRs are either ordered prior to involving g-GPA or the ordering is left to any of the g-GPAs. Note that for the GPA algorithm, ordering of subchannels does not led to any improvement in complexity as search step (4) in the while loop—which represents the bottleneck of the overall computations—has to include all subchannels. This is due to the fact that by relaxing the grouping concept it is possible to find subchannels in lower QAM levels that need less power to upgrade than others in higher QAM levels, whereas in the case of the g-GPA algorithm, initial sorting of subchannels according to their CNR_*i*_ is sufficient to avoid the repetitive search/sorting step (3) of Algorithm 2 as this algorithm is independently applied to subchannels that are bounded by one QAM level only.

The quantities *L*_1_ and *L*_2_^*k*^ in Table 1 denote, respectively, the averaged number of iterations of the while loops for the GPA algorithm in Algorithm 1 and the g-GPA algorithm in Algorithm 2. Note that it is expected that *L*_1_ ≥ *L*_2_ = ∑_*k*=0_^*K*−1^ *L*_2_^*k*^ as *P* ^ex^ in (11) collected from all subchannels has to be redistributed by the GPA algorithm, while *P*_*k*_^ex^ collected from only subchannels *i* ∈ G_*k*_ is considered by the g-GPA algorithm.

Obviously, *N*_*k*_ in (11) cannot be easily quantified as it depends on both the operating SNR and CNR_*i*_, which for Rayleigh channels is a chi-squared distributed random variable. Therefore, the complexity of g-GPA is evaluated in a heuristic fashion. In the worst case and by assuming that subchannels are uniformly distributed across all QAM groups, that is, *N*_*k*_ = *N*/*K*, the complexity of the g-GPA algorithm can be approximated as given in Table 1 which is lower than its GPA counterpart.

## 5. Simulation Results and Discussion

Sections 3.2 and 3.3 have shown that both Mu-GPA and Md-GPA algorithms work very similarly in utilising the power *P*_*k*_^LO^ that remains unused after applying the g-GPA algorithm to all groups *k*,  0 ≤ *k* ≤ *K* − 1. The two algorithms differ in the direction in which *P*_*k*_^LO^ is transferred. Below we compare by simulations the bit allocation performance of the two algorithms with the UPA, GPA, and g-GPA approaches. Two sets of simulations are conducted to explore the achieved data throughput of the considered algorithms for the case of narrowband MIMO and OFDM-multicarrier systems, whereby the latter is characterised by a much higher number of subchannels.

### 5.1. Narrowband MIMO Case

The proposed loading schemes are first tested on a 4 × 4 narrowband MIMO system to investigate bit loading performance. The entries of the channel matrix **H** are drawn from a complex Gaussian distribution with zero mean and unit variance; that is, *h*_*ij*_ ∈ *𝒞𝒩*(0,1). The subchannels are obtained by means of an SVD, which provides optimal joint linear precoding and equalisation in a number of senses [15] and yields subchannel gains that are equivalent to the 4 singular values of **H**. Results presented below refer to ensemble averages across 10^4^ different channel realisations for a target BER of *𝒫*_*b*_^target^ = 10^−3^ and various levels of SNRs using QAM modulation schemes *M*_*k*_ = 2^*k*^, *k* = 1 ⋯ *K*, with *K* = 6 being the maximum permissible QAM level with constellation size *M*_*K*_ = 64, which is equivalent to encoding 6 bits per data symbol.

**Figure 4:**
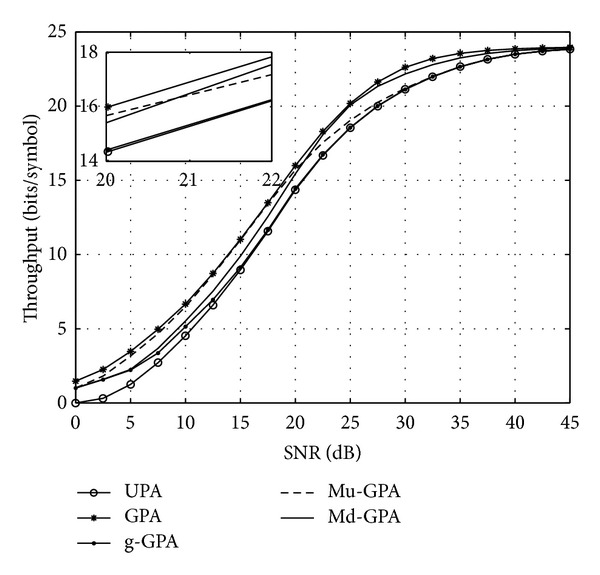
Overall throughput for a 4 × 4 MIMO system with a target BER of *𝒫*_*b*_^target^ = 10^−3^.

The total system throughput is examined and shown in Figure 4 for all proposed algorithms in addition to both UPA and standard GPA algorithms. It is evident that UPA represents an inefficient way of bit loading since the performance is approximately 2 to 5 dB below other algorithms when operating under moderate SNRs between 10 and 30 dB, and provides approximately only half the throughput in the SNR region between 5 and 10 dB.

Of the proposed low-cost greedy algorithms, both Mu-GPA and Md-GPA algorithms outperform the g-GPA without the refinement stage to allocate residual power across QAM groups. Interestingly, Mu-GPA performs better at low SNR, while Md-GPA performs better at higher SNRs. This can be attributed to the fact that, for low-to-medium SNRs, *P*_*K*_^ex^ (which is missed by the Mu-GPA) will be relatively low and can be allocated without violating the constraint on the maximum QAM level *M*_*K*_. In contrast, *P*_0_^ex^, which is missed by the Md-GPA, is most likely to be high as evident from (11) and Figure 1. For medium-to-high SNRs, *P*_*K*_^ex^ > *P*_0_^ex^ can be expected to be high, and thus Md-GPA is likely to be advantageous in its bit allocation, as the maximum QAM level constraint is beginning to be felt and *P*_*K*_^ex^ is fully utilised by the Md-GPA algorithm.

Finally, for very high SNRs most subchannels will appear in the highest QAM group G_*K*_ as their SNRs, *γ*_*i*_ in (8), exceed the highest QAM level *γ*_*K*_^QAM^ in (6). As a result, the overall system throughput of all different algorithms reaches its expected maximum of 4 × *b* ^max⁡^ bits/symbol.

**Figure 5:**
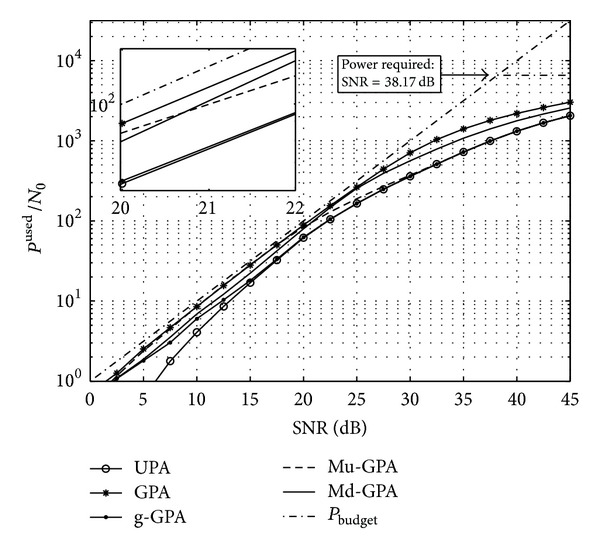
Power used by the considered algorithms for a 4 × 4 MIMO system to achieve their respective throughput in Figure 4.

The data throughput performance of the various algorithms can also be confirmed when considering the power utilisation.  Figure 5 shows the total transmit power budget and the levels of power allocation that are reached by the different algorithms. For Md-GPA and Mu-GPA it can be noted that, within their respective superiority regions, both are very close to the performance of the standard GPA which demonstrates the efficient utilisation of the LO power missed by the g-GPA algorithm. Nevertheless, at high SNR, both g-GPA and Mu-GPA algorithms behave like the UPA algorithm due to the increase of *P*_*K*_^ex^, which is missed by both of them and therefore deteriorates their performances. Note that the minimum theoretical transmit power that according to (6) is required to load all subchannels with *b* ^max⁡^ averaged over all 10^4^ channel realisations corresponds to an approximate SNR of 38.17 dB as shown in Figure 5.

### 5.2. OFDM-Multicarrier Case

Another simulation set is conducted to examine the performance of our proposed schemes for an OFDM-multicarrier system with a significantly higher number of subchannels as considered in Section 5.1. Here we assume a SISO OFDM system, whereby the ISI channel is characterised by an impulse response vector **h** of order *L* = 6 with entries drawn from an independent complex Gaussian process with zero mean and unit variance. Results are conducted for a 32-subcarrier system averaged over 10^4^ channel realisations for a target BER of *𝒫*_*b*_^target^ = 10^−3^ and varying SNR using the same QAM modulation schemes as in Section 5.1.

**Figure 6:**
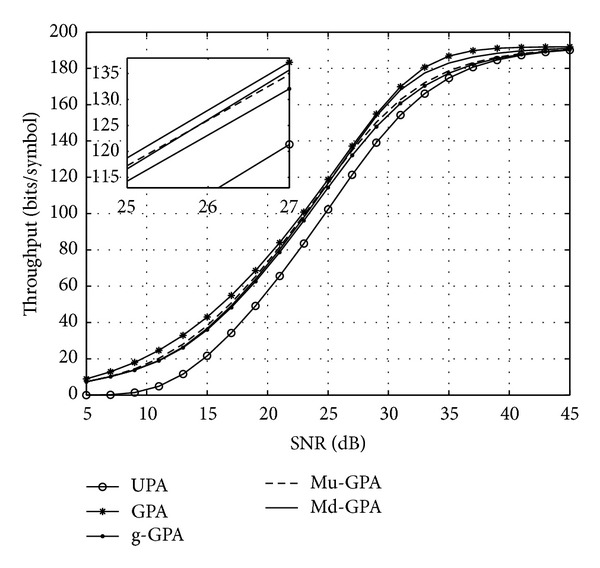
Overall throughput for a 32-subcarrier system with a target BER of *𝒫*_*b*_^target^ = 10^−3^.

**Figure 7:**
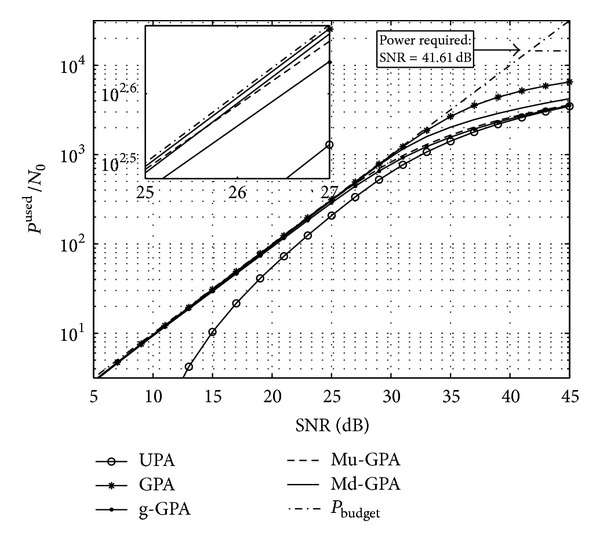
Power used by the considered algorithms for a 32-subcarrier system to achieve their respective throughput in Figure 6.

The total system throughput is shown in Figure 6 for all proposed algorithms in addition to both UPA and standard GPA algorithms. It is clearly shown that both Mu-GPA and Md-GPA algorithms perform very close to the GPA algorithm (with throughput loss ≤4 bits) within their SNR favourable regions, which swap approximately at SNR = 25.82 dB.  Figure 7 again shows the power usage of all algorithms that is required to reach their respective throughput in Figure 6. Compared to the optimum GPA, the Md-GPA algorithm demonstrates very similar power utilisation with some inferior performance due to missing to allocate the final LO power in (18). At higher SNRs, both Mu-GPA and g-GPA algorithms converge to the power usage performance of the UPA algorithm as *P*_*K*_^ex^ dominates other *P*_*k*_^ex^, 0 ≤ *k* ≤ *k* − 1, and therefore only the Md-GPA algorithm is advantageous in this region. The minimum theoretical transmit power required to load all subcarriers with *b* ^max⁡^ in this case is shown to be equivalent to an approximate SNR of 41.61 dB.

### 5.3. Computational Complexity Results

In order to evaluate the computational complexity of the proposed scheme compared to the standard GPA algorithm, the number of algorithmic operations presented in the complexity analysis in Section 4 is tested and compared for both g-GPA and GPA algorithms using a 1024-subcarrier system.  Table 2 gives the simulation results of the number of operations—averaged over 10^4^ channel instances—for both “no order” and “order” cases of the g-GPA algorithm along with the GPA algorithm at three different values of SNR of 15 dB, 25 dB, and 35 dB. Note that *L*_2_ = ∑_*k*=0_^*K*−1^ *L*_2_^*k*^ is less than *L*_1_ for all SNR values which validates the complexity analysis of Section 4. Furthermore, a reduction of almost half the number of operations can be gained by ordering subchannels of the g-GPA algorithm, which results in an overall reduction factor compared to the full GPA algorithm of approximately 2, 3, and an order of magnitude for the considered SNR values, respectively (cf.  Table 2).

**Figure 8:**
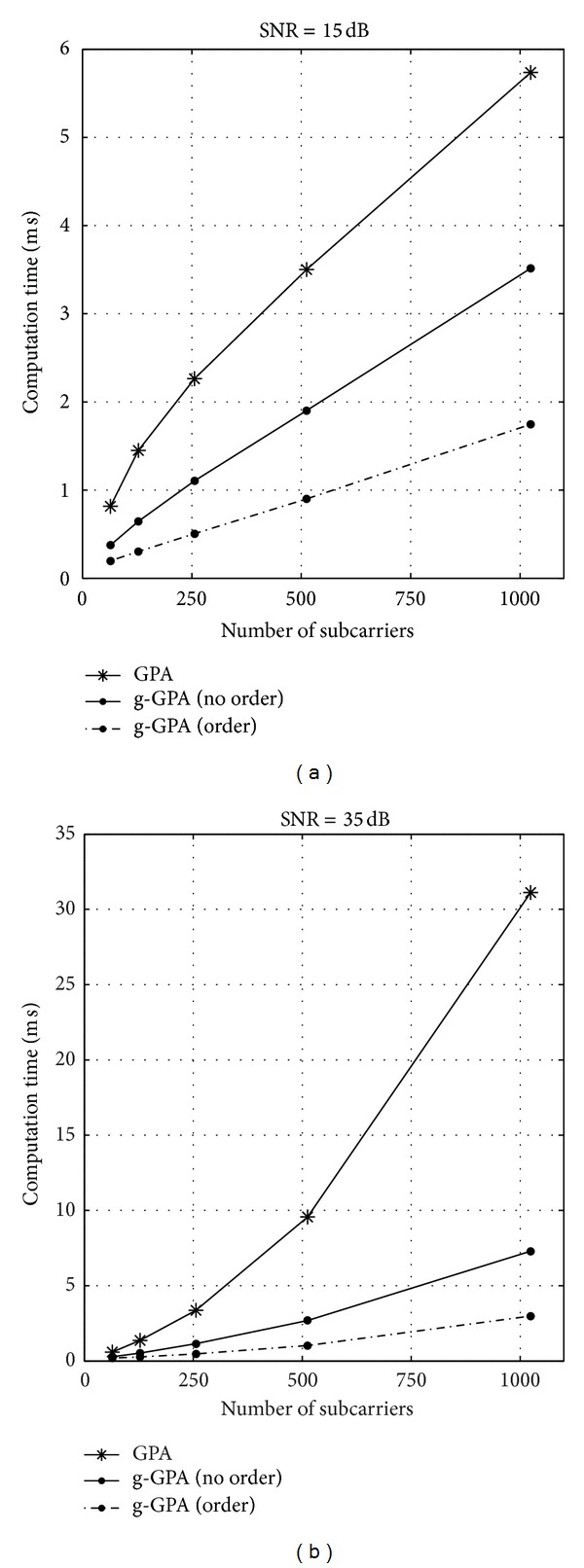
Average computation time comparison of the g-GPA and the GPA algorithms for *𝒫*_b_^target^ = 10^−3^ and varying *N*-subcarrier system at different SNR applications for (a) 15 dB SNR and (b) 35 dB SNR.

The complexity analysis can also be evaluated by investigating the computation time of both GPA and g-GPA algorithms.  Figure 8 shows the computation time against the number of subcarriers *N* for the g-GPA algorithm with both “no order” and “order” cases compared to the GPA algorithm. Two different SNRs values of 15 dB and 35 dB that represent the approximate conditions of mobile and fixed wireless communication, respectively, are considered in this simulation. It is clear that the g-GPA algorithm has a higher computational efficiency in particular for large values of *N* and high SNRs, while the effect of subcarrier ordering is also evident as discussed in Section 4. Assuming a close correlation between the number of operations and their computation time, it is noted that at *N* = 1024 subcarriers these results coincide with that of Table 2.

**Figure 9:**
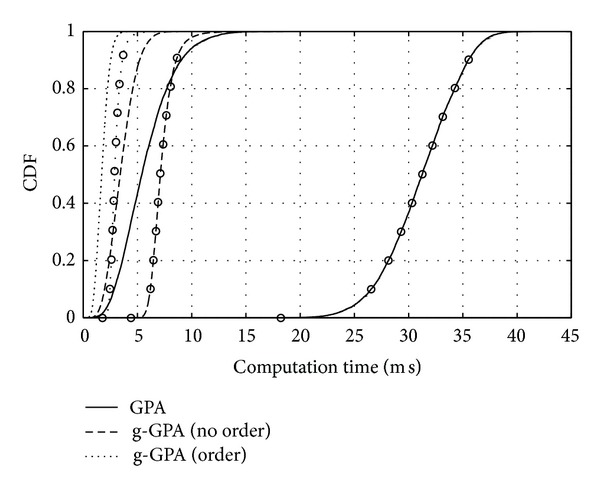
Cumulative distribution function of the computation time for a 1024-subcarrier system and *𝒫*_*b*_^target^ = 10^−3^ at SNR values of 15 dB (without circles) and 35 dB (with circles).

In a statistical fashion, Figure 9 demonstrates the cumulative distribution function (CDF) of the computation time for both algorithms at the same SNR values which reveals the computational efficiency of the proposed g-GPA algorithm and its modified versions of both Mu-GPA and Md-GPA.

## 6. Conclusions

Power allocation to achieve maximum data throughput under constraints on the transmit power and the maximum QAM level has been discussed. The optimum solution is provided by the greedy power allocation (GPA) algorithm, which operates across all subchannels but is computationally very expensive. Therefore, in this paper suboptimal low-complexity alternatives have been explored. The common theme amongst the proposed algorithms is to restrict the GPA algorithm to subsets of subchannels, which are grouped according to the QAM levels assigned to them in the uniform power allocation stage. In order to exploit excess (unused) power in each subset, two algorithms were created which carry left-over power forward into the next subset that is optimised by a local greedy algorithm. Two different schemes have been suggested, of which one moves the left-over power upwards from the lowest to the highest subgroup, where in the high SNR case a limitation by the maximum defined QAM level can restrict the performance. A second scheme moves the power from the highest towards the lower subgroups, whereby at low SNR the channel quality in the lowest subgroups may not be such that it can be lifted across the lowest QAM level, and hence no bits may be loaded with the excess power. However, in general both algorithms perform very close to the GPA in their respective domains of preferred operation, thus permitting to allocate power close to the performance of the GPA at a much reduced cost.

## Figures and Tables

**Algorithm 1:**
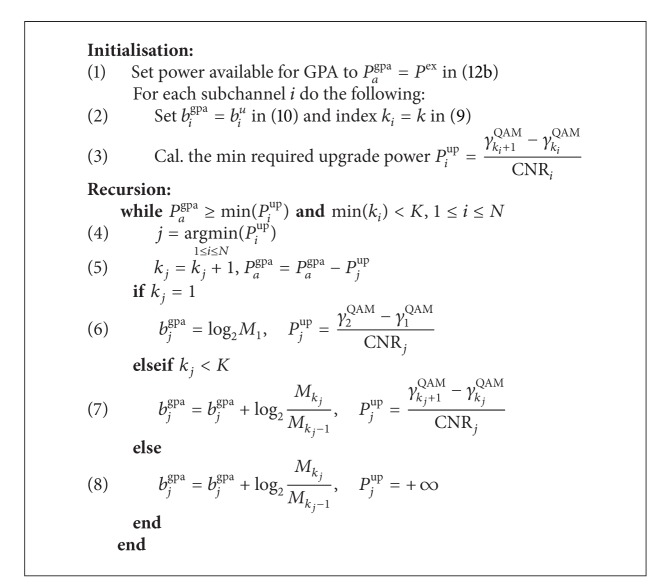
Full GPA algorithm applied to the initialisation step of the UPA algorithm.

**Algorithm 2:**
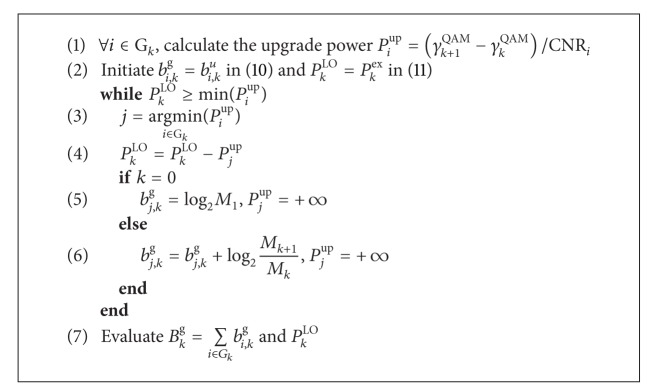
g-GPA algorithm for subchannels in the *k*th QAM group G_*k*_.

**Table 1:** Computational analysis for both GPA and g-GPA algorithms.

Algorithm	Number of operations (NoO)
GPA	*L*_1_(2*N* + 7) + 4*N* + 1

g-GPA (no order)	$\overset{K-1}{\underset{k=0}{\sum }}L_{2}^{k}(2N_{k}+4)+2N_{k}+2\approx L_{2}\left(2\frac{N}{K}+4\right)+2\frac{N}{K}+2$
g-GPA (order)	$\overset{K-1}{\underset{k=0}{\sum }}L_{2}^{k}(N_{k}+5)+2N_{k}+2\approx L_{2}\left(\frac{N}{K}+5\right)+2\frac{N}{K}+2$

**Table 2:** Simulation results for the parametric analysis of the GPA and g-GPA algorithms given in Table 1 for a 1024-subcarrier system and different SNR values.

Algorithm: GPA						
SNR	15 dB		25 dB		35 dB	

*L*_1_	112.5		600.6		621.2	
NoO × 10^3^	**235.4**		**1,238.3**		**1,280.6**	

Algorithm: g-GPA						
QAM groups	*N*_*k*_	*L*_2_^*k*^	*N*_*k*_	*L*_2_^*k*^	*N*_*k*_	*L*_2_^*k*^

G_0_	1,024	103.2	946.8	425.1	234.7	140.6
G_1_	0	0	71.5	23.1	178.2	89.6
G_2_	0	0	5.7	0.89	293.0	140.2
G_3_	0	0	0	0	229.5	96.7
G_4_	0	0	0	0	80.9	27.0
G_5_	0	0	0	0	7.7	1.55
NoO × 10^3^ (no order)	**213.8**		**812.2**		**232.8**	
NoO × 10^3^ (order)	**108.3**		**408.5**		**118.9**	
